# New System of Shrinkage Measurement through Cement Mortars Drying

**DOI:** 10.3390/s17030522

**Published:** 2017-03-06

**Authors:** Carlos Morón, Pablo Saiz, Daniel Ferrández, Luisa García-Fuentevilla

**Affiliations:** 1 Sensors and Actuators Group, Department of Building Technology, Madrid Technical University, 28040 Madrid, Spain; daniel.ferrandez.vega@alumnos.upm.es (D.F.); luisa.garcia.fuentevilla@alumnos.upm.es (L.G.-F.); 2 Architectural Construction and Control Department, Madrid Technical University, 28040 Madrid, Spain; pablo.saiz@upm.es

**Keywords:** capacitive sensor, moisture, shrinkage, cement mortar, recycled aggregate

## Abstract

Cement mortar is used as a conglomerate in the majority of construction work. There are multiple variants of cement according to the type of aggregate used in its fabrication. One of the major problems that occurs while working with this type of material is the excessive loss of moisture during cement hydration (setting and hardening), known as shrinkage, which provokes a great number of construction pathologies that are difficult to repair. In this way, the design of a new sensor able to measure the moisture loss of mortars at different age levels is useful to establish long-term predictions concerning mortar mass volume loss. The purpose of this research is the design and fabrication of a new capacitive sensor able to measure the moisture of mortars and to relate it with the shrinkage.

## 1. Introduction

Massive exploitation of natural recourses in the Spanish construction sector increased in the years prior to the economic crisis with the exponential construction of new buildings, considerably affecting the environment. Under these circumstances, the current normative framework places emphasis on the incorporation of a new recycling politics, reducing in this way the use of natural raw material and allowing more sustainable development of the construction sector. Such is the case of the current Instruction on Structural Concrete EHE-08 that contains the specifications for recycled aggregates to be used in concretes in one of its annexes [1]. Construction and demolition waste (CDW) is produced during the construction and infrastructure life cycle, and in the rehabilitation and demolition processes [2]. CDW being correctly treated in recycling plants can be used as a material for the construction of new buildings, as is the case with ceramic and concrete recycled aggregates used as a raw material in mortars fabrication [3,4]. Nevertheless, the average recycling rate in Spain (15%) is much lower than the European average (50%), whereas some of the countries such as Denmark and Holland recycle more than 90% of their CDW [5].

In terms of the properties of recycled aggregates, there are many authors who studied their physical, chemical, and mechanical characteristics for their use in the construction, which are generally affected when the percentage of fine recycled aggregate replacement is more than 25% of aggregate content, producing mortars with lower density and higher water absorption [6].

In terms of the contribution of ceramic aggregates in mortar production, they did not significantly affect the mechanical behaviour of mortar when the percentage of ceramic recycled aggregate was not higher than 40% of the total volume of the natural aggregate; what is not true with other properties, such as density and workability [7]. Nevertheless, some authors replaced 100% of natural aggregate with ceramic recycled aggregate, showing that it is possible to reach excellent behaviour with an adequate fabrication process of these mortars [8,9]. On the contrary, other authors observed that the contribution of ceramic recycled aggregates improves bond strength between them and cement paste due to their form, which is less rounded compared to natural aggregates [10]. 

On the other hand, the use of concrete recycled aggregate in cement mortars fabrication has negative consequences in terms of durability, as in general they are less strong and have a major pore content [11]. Due to this fact, the great majority of current studies fix the maximum percentages of concrete recycled aggregates in mortar mix to obtain reliable mixes with properties similar to those observed in natural aggregates [12,13]. 

One of the properties that is seen to be more affected in mortars fabricated with recycled aggregates is shrinkage, because the water absorption of this type of aggregate is much higher than that of natural aggregates [14]. This phenomenon of drying shrinkage is associated with bond strength loss in mortars, which may cause easier mortar paste detachment [15], caused by the volume reduction of mortars through the evaporation of water during its hydration [16]. For this reason, this phenomenon depends on various factors, such as drying ambient temperature, relative humidity of air, size and form of the specimen, the relation water–cement, and the relation cement–aggregate [17]. 

Thus, higher shrinkage is related to greater variation in the content of the specimen that can be measured using specific experimental equipment. 

Nevertheless, existing methodology of shrinkage measure through drying that was developed for cement mortars presents serious problems in its application to mortars fabricated with recycled aggregates, due to different hardening mechanisms. Nonetheless, because shrinkage is related to moisture variation, electronic equipment can be used to measure this variation of water content, which is more effective than traditional gravimetric methods based on the progressive loss of weight until reaching anhydrous state [18]. To this end, some authors used strain sensors based on the conductivity of two terminals to determine water content, obtaining good results in the measure of the moisture level of soils and its application in automatic irrigation systems [19]. Ultrasonic moisture meters based on different propagation speeds of waves that go through certain types of material were also used. In general, the application of ultrasounds in mortar and concrete is delegated to initial inspections, being a less precise measure of moisture content [20]. In this field of material moisture measure, capacitive sensors are very well accepted due to their easy fabrication, application, and low cost compared to other typologies. Some authors showed that application range of these sensors can be very wide, around 175–625 ppm [21], enabling their application in different materials; e.g., measurement of the moisture level and degradation of wood [22] and moisture content measurement in gases using high linearity in the capacitive sensors’ response [23]. This ideal/linear response is not always possible to obtain; such is the case with some strain sensors used to measure deformations and study material microstructure [24], whose response should be studied and improved to be interpreted. Due to its high sensibility, another application of the capacitive sensors is the monitoring of the water content in industrial fabrication lines [25].

Thus, the aim of this research is the design and fabrication of a new measuring equipment based on a capacitive sensor that allows a response to be obtained that can be related to moisture content of the specimens of mortar at different age levels, in order to extrapolate the results of shrinkage that these specimens experiment according to their volume loss. In such a way, the results of this research can improve the precision given by the current method of shrinkage measure through drying in mortars based on the measure of specimen length at different age levels. 

## 2. Methodology

This section describes both the process of fabrication of cement mortar specimens and the design of the sensor, as well as methods of moisture and shrinkage measure that were used. 

### 2.1. Fabrication of Mortar Specimens

The codification that was used to identify different mortar mixes is the following: M-N-D, where M is a masonry mortar; N is a type of used aggregate that can be natural aggregate (NA), concrete recycled aggregate (Con RA) or ceramic recycled aggregate (Cer RA); D is a cement-aggregate in weight proportion, where X is used for 1:3 proportion and Y is used for 1:4 proportion.

The dosage in weight used for different specimens can be observed in Table 1, where used quantities and ratios are indicated.

The fabrication of all the mixes was carried out using the same technique and equipment according to the requirements of the standard UNE-EN 196-1 [26]. Cement mortar mixes were elaborated using a planetary mixer CIB-701 model of Ibertest brand with 3 litre capacity. Firstly, the aggregates, cement, and water were weighted separately using a scale of precision to 0.01 gram. Then, water and cement were poured into the bowl of the planetary mixer, while the aggregates were put into the hopper of the mixer. The time used for mortar mixes elaboration was fixed by the above-mentioned standard. In the mixes fabricated with recycled aggregates, it was necessary to use the plasticizer Glenium Sky 604, which is recommended for use in mortars by the BASF Company. This additive was incorporated in 1% over the weight of cement, complying with the recommendations of the manufacturer in order to obtain proper consistency. In this way, all the mixes elaborated in this work are of plastic consistency according to the values of 175 ± 10 mm established by the standard UNE-EN 1015-3 [27]. The dimension of the specimens was 25 × 25 × 287 mm. 

### 2.2. Capacitive Sensor Design

**Figure 1:**
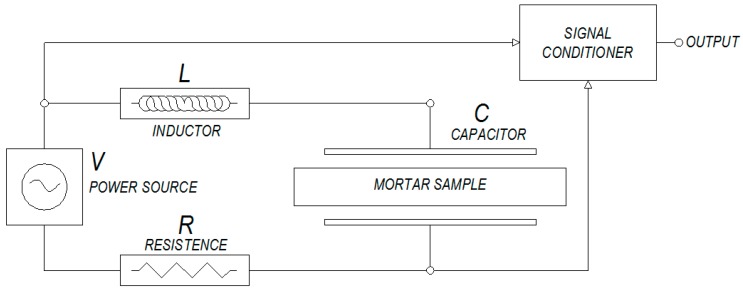
Capacitive sensor scheme.

A capacitive sensor was developed to measure the moisture of mortar specimens. This sensor is connected to the auto-oscillatory circuit able to measure light variations in water loss of mortar specimens. The scheme of the used sensor can be observed in Figure 1. 

As can be seen in Figure 1, the sensor consists of two flat parallel sheets of cooper, whose surface dimensions are 25 × 247 mm adapting to the surface of samples without including their edges. 

The functioning principle of the sensor is based on the measure of capacitance of the condenser as the changes of moisture produced by water loss modify the permittivity of the mortar that acts as a dielectric according to Equation (1):

(1) $$C=\epsilon \cdot \frac{A}{d}\;\left[F\right]$$

where *C* is the capacitance of the condenser, ϵ is the permittivity of the dielectric (in this case of wet sample of mortar), A is the surface of condenser sheets, and d is the distance between the sheets (in this case, it coincides with sample thickness).

In such a way, if the surface and separation between sheets are maintained fixed, the variation in the capacitance of the sensor will depend only on the variation of the permittivity of a dielectric or on the moisture content of the sample. 

Moreover, the resonance frequency of the auto-oscillatory circuit is directly related to the capacitance of the sensor and the inductance according to Equation (2):

(2) $$f=\frac{1}{\sqrt{LC}}\;\left[Hz\right]$$

where *f* is the resonance frequency, *C* is the capacitance determined by Equation (1), and *L* is the induction of the reel. 

In this way, it is possible to relate moisture loss of mortar samples after being mixed and their shrinkage to the capacitance value of the used sensor.

### 2.3. Method of Moisture and Shrinkage Measure

After being prepared, the mortar samples were left in the laboratory under constant conditions of temperature of 20 ± 1 °C and moisture of 60% ± 2%. Once setting began, the process of hydraulic shrinkage was observed due to the evaporation of excess water in the mix that stays inside the sample without combination. Equation (3) models this type of shrinkage:

(3) $$R_{H}=2R_{M}\cdot \frac{{\left(1-\eta \right)}^{0.7}}{D^{n}}\cdot \left[1-\frac{1}{{\left(1+0.79{\left(\frac{s}{v}\right)}^{2}t\right)}^{0.2}\cdot 1.024^{\;\left(\frac{s}{v}\right)2t}}\right]$$

where $R_{H}$ is hydraulic shrinkage, $R_{M}$ is maximum shrinkage in about one year, η is relative humidity in the atmosphere, D is maximum particle size of aggregate in mm, n is a variable that depends on the holes left by aggregate, s/v is the free surface divided by the volume of mortar, and *t* is time in days.

The traditional method of measuring shrinkage in mortar samples is established by the standard UNE 80-112-89 [28], and consists of the measurement of the length of samples using a length comparator once samples are unmolded. Thus, shrinkage is expressed as a percentage $\Delta L_{nd}\left(\text{\%}\right)$ with respect to the initial length given by Equation (4):

(4) $$\Delta L_{nd}=\frac{M_{nd}-M_{1d}}{L_{0}}\cdot 100\;\left[\%\right]$$

where $L_{0}$ is the base length of mould, $M_{nd}$ is a sample measure with the comparator in *n* days, and $M_{1d}$ is a measure of sample with the comparator the first day. 

Thus, shrinkage is determined by the volume loss of mortar once setting starts. On the other hand, the moisture content can be determined through the process of weighting according to Equation (5):

(5) $$M=\frac{W_{nd}-W_{0}}{W_{0}}\cdot 100\;\left[\%\right]$$

where M is moisture expressed in percentage, $W_{nd}$ is the weight of sample on day *n*, and $W_{0}$ is the weight of sample in anhydrous state. 

Finally, after being measured and weighted, the samples are kept in the laboratory under constant temperature and humidity to avoid any possible influence on the measures of the sensor, obtaining values of capacitance for each sample in order to establish a correlation between the methods of moisture measurement and shrinkage measured during the period of time of 128 days. 

**Figure 2:**
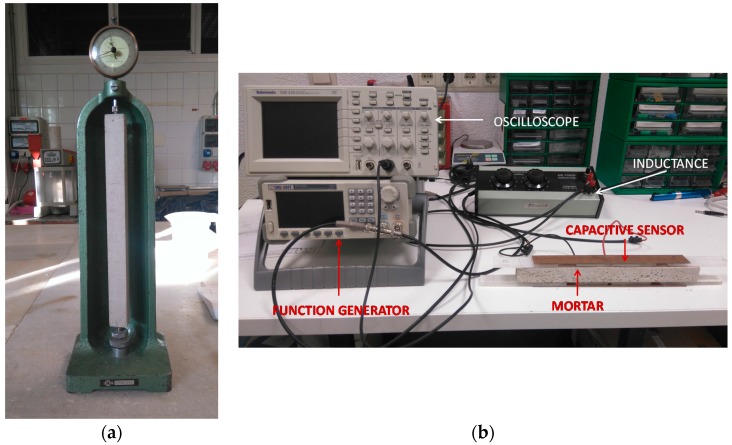
Measuring equipment. (**a**) Manual device of shrinkage measure; (**b**) Capacitive sensor and auto-oscillatory circuit for moisture loss measure.

Figure 2 shows the manual shrinkage measuring machine based on volume loss described before and the system based on the measure of capacitance of the sensor. 

## 3. Results and Discussion

This part shows the results obtained from the measurement of three variables: moisture, capacitance, and shrinkage, establishing a correlation between them for different dosages. 

### 3.1. Variation of Capacitance with Regard to the Moisture

Firstly, the results obtained using the sensor for the measurement of moisture in mortar samples are shown. 

**Figure 3:**
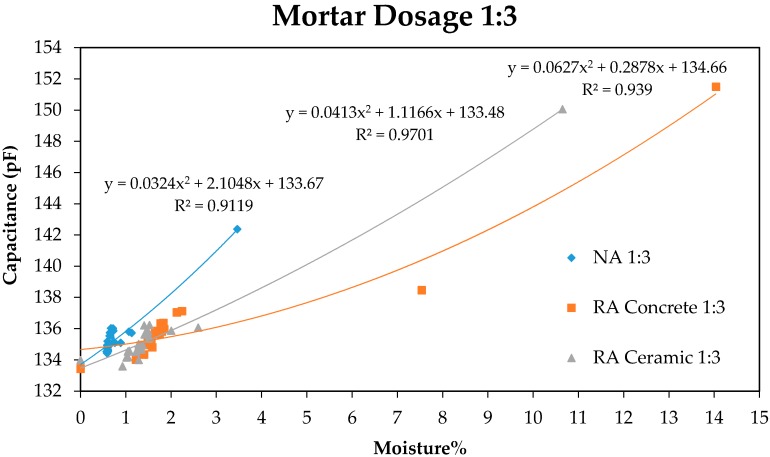
Relation Moisture–Capacitance for mortars with 1:3 dosage.

**Figure 4:**
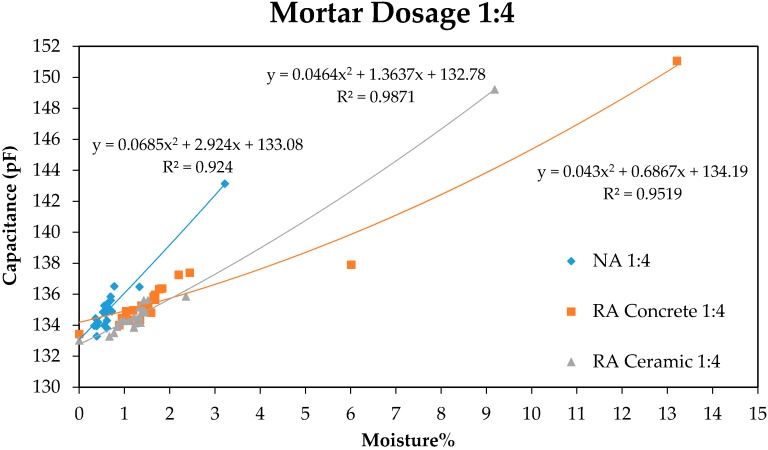
Relation Moisture–Capacitance for mortars with 1:4 dosage.

In Figure 3 and Figure 4, a good correlation between the response in pF of the capacitive sensor and the percentage of moisture in mortar samples can be observed. 

Figure 3 and Figure 4 show that the variation of the capacity of the condenser with regard to moisture loss in mortar samples follows a parabolic behaviour according to the equations presented in the graphics, obtained by the process of adjusting through the method of least squares. The last measure that corresponds to 0% moisture was carried out by taking the samples to anhydrous state through drying in a laboratory oven. On the other hand, it can be observed that during the first days the moisture loss is much more important, a lower density of points existing between the first and the last measures. 

Moreover, a great difference in the response of the sensor between the first and the second measure can be observed. This is caused by the strong exothermal reaction produced in the mortars after setting, where a great part of mix water combines with cement that conforms mortar, releasing a great quantity of energy that facilitates the evaporation of water that stays inside the sample without combination. In contrast, the variation of moisture from this moment onwards is less pronounced, in accordance with the theoretical predictions. 

### 3.2. Variation of Shrinkage with Regard to the Moisture

**Figure 5:**
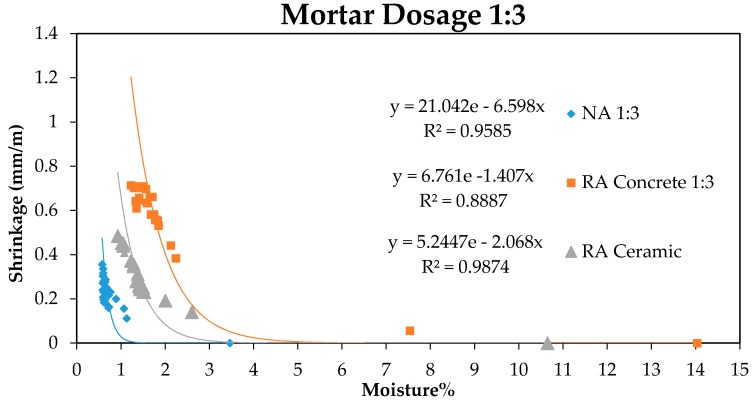
Relation Moisture–Shrinkage for mortars with 1:3 dosage.

**Figure 6:**
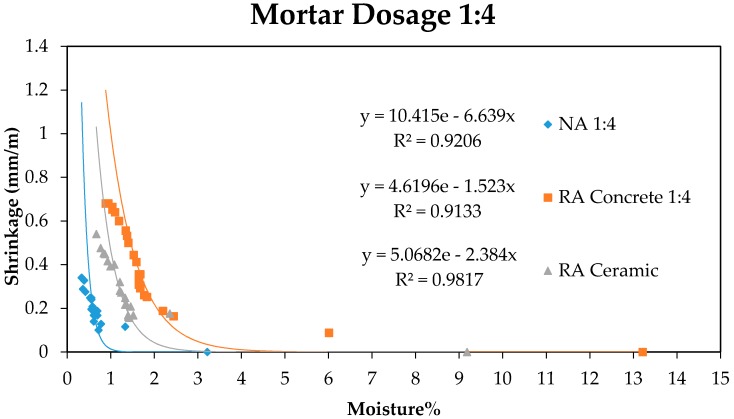
Relation Moisture–Shrinkage for mortars with 1:4 dosage.

Furthermore, a relation between the moisture loss of samples and manually measured shrinkage was established. The obtained results are shown in Figure 5 and Figure 6. 

As can be observed in Figure 5 and Figure 6, the moisture loss produces a shortening in the initial length of the sample as age increases, following the exponential behaviour observed in the graphics. This shrinkage related to mortar drying is more pronounced in recycled aggregates compared to natural aggregates, in accordance with the values of capacity measured with the sensor in Figure 3 and Figure 4. In such a way it is possible to establish a relation between mortar shrinkage values that were expected with the measures obtained using the capacitive sensor, as both measures depend on samples’ moisture loss. 

## 4. Conclusions

A system that allows the moisture content of samples to be obtained was developed. For the case of moisture loss in cement mortars, the capacitance follows a parabolic behaviour regarding moisture content, these values being more pronounced when the quantity of water inside the sample increases. 

Moreover, an exponential relation between the moisture content and drying shrinkage was observed. A direct relation between the response in capacity of the sensor and a sample volume loss was obtained comparing both measures, thus validating this method as an alternative measurement method for drying shrinkage in cement mortars. 

## Figures and Tables

**Table 1:** Mortars dosages.

Type	Cement (g)	Aggregate (g)	Water (g)	Additive (%)	Proportion	
					(c/a) ^2^	(w/c) ^3^
*M-NA-X*	450	1350	261	-	1:3	0.58
*M-Con RA-X*	450	1350	306	1 ^1^	1:3	0.68
*M-Cer RA-X*	450	1350	306	1 ^1^	1:3	0.68
M-NA-Y	337.5	1350	202.5	-	1:4	0.60
*M-Con RA-Y*	337.5	1350	300.4	1 ^1^	1:4	0.89
*M-Cer RA-Y*	337.5	1350	300.4	1 ^1^	1:4	0.89

Note: ^1^ 1% of additive over the weight of cement; ^2^ Cement–aggregate dry proportion; ^3^ Water–cement proportion. Cer RA: ceramic recycled aggregate; Con RA: concrete recycled aggregate; M: masonry mortar; NA: natural aggregate; X: 1:3 cement to aggregate weight ratio; Y: 1:4 cement to aggregate weight ratio.
